# Supplementary material for: Therapeutic Potential of Exosomes in Tendon and Tendon–Bone Healing: A Systematic Review of Preclinical Studies
Source: J Funct Biomater. 2023 May 28;14(6):299. doi: 10.3390/jfb14060299 (PMC10299056; doi:10.3390/jfb14060299)
Supplement: Supplementary file 1 [file jfb-14-00299-s001.zip › Table S2~Table S7.pdf]

Table S2: Specific search Strategy

**1**	Exosome* (Title OR Abstract OR Key words)
**2**	Extracellular vesicle* (Title OR Abstract OR Key words)
**3**	Exosomal (Title OR Abstract OR Key words)
**4**	Cell-Derived Microparticle* (Title OR Abstract OR Key words)
**5**	Secretome* (Title OR Abstract OR Key words)
**6**	Tendon* (Title OR Abstract OR Key words)
**7**	Ligament* (Title OR Abstract OR Key words)
**8**	Rotator cuff (Title OR Abstract OR Key words)
**9**	Achilles (Title OR Abstract OR Key words)
**10**	Enthesis (Title OR Abstract OR Key words)
**11**	Footprint (Title OR Abstract OR Key words)
**12**	Tendinous* (Title OR Abstract OR Key words)
**13**	Tendinopathy (Title OR Abstract OR Key words)
**14**	Tendinopathies (Title OR Abstract OR Key words)
**15**	Tendinosis (Title OR Abstract OR Key words)
**16**	Tendinitis (Title OR Abstract OR Key words)
**17**	1 OR 2 OR 3 OR 4 OR 5
**18**	6 OR 7 OR 8 OR 9 OR 10 OR 11 OR 12 OR 13 OR 14 OR 15 OR 16
**19**	17 AND 18

Table S3: Inclusion and Exclusion Criteria

	**Inclusion Criteria**	**Exclusion Criteria**
**Study type**	Original Article	Patent<br>Conference Abstract<br>Letter<br>Case report<br>Editorial and Editorial materials<br>Review article
**Language**	English	Not English
**Topic**	Exosome and Tendon healing<br>Exosome and Tendon-bone healing	Periodontal ligament or dental related diseases<br>Not related to tendon OR tendon-bone healing<br>Not related to Exosomes
**Experiment type**	In vivo or ex vivo experiment<br>In vivo (ex vivo) and in vitro experiment	Only with in vitro experiment
**Quality**	Good quality of research studies	Poor quality of research

For articles which focus on the effect of extracellular vesicles promoting tendon, tendon-bone or ligament repair, we examined the distribution of extracellular vesicle diameters. Articles in which the distribution of diameters were significantly outside the distribution of exosomes (30~150nm) were excluded.

Table S4: Risk of bias assessed using SYRCLE risk of bias assessment tool

Author	Year	Selection bias			Performance bias		Detection bias		Attrition bias	Reporting bias	Other bias
		Sequence generation	Baseline Characteristics	Allocation Concealment	Random housing	Blinding	Random Outcome assessment	Blinding	Incomplete outcome data	Selective outcome reporting	Other sources of bias
Chamberlain et.al[1]	2019	Unclear risk	Low risk	Unclear risk	Unclear risk	Unclear risk	Low risk	Unclear risk	Low risk	Low risk	Low risk
Chen et.al[2]	2021	Unclear risk	Low risk	Unclear risk	Unclear risk	Unclear risk	Low risk	Unclear risk	Low risk	Low risk	Low risk
Cui et.al[3]	2019	Unclear risk	Low risk	Unclear risk	Unclear risk	Unclear risk	Low risk	Low risk	Low risk	Low risk	Low risk
Davies et.al[4]	2022	Unclear risk	Low risk	Unclear risk	Unclear risk	Unclear risk	Low risk	Unclear risk	Low risk	Low risk	Low risk
Fu et.al[5]	2021	Unclear risk	Low risk	Unclear risk	Unclear risk	Unclear risk	Low risk	Unclear risk	Low risk	Low risk	Low risk
Gao et.al[6]	2022	Unclear risk	Low risk	Unclear risk	Unclear risk	Unclear risk	Low risk	Unclear risk	Low risk	Low risk	Low risk
Gissi et.al[7]	2020	Unclear risk	Low risk	Unclear risk	Low risk	Unclear risk	Low risk	Low risk	Low risk	Low risk	Low risk
Han et.al[8]	2022	Unclear risk	Low risk	Unclear risk	Unclear risk	Unclear risk	Low risk	Low risk	Low risk	Low risk	Low risk
Han et.al[9]	2022	Unclear risk	Low risk	Unclear risk	Unclear risk	Unclear risk	Low risk	Unclear risk	Low risk	Low risk	Low risk
Hayashi et.al[10]	2022	Unclear risk	Low risk	Unclear risk	Low risk	Unclear risk	Low risk	Low risk	Low risk	Low risk	Low risk
Huang et.al[11]	2020	Unclear risk	Low risk	Unclear risk	Unclear risk	Unclear risk	Low risk	Unclear risk	Low risk	Low risk	Low risk
Jenner et.al[12]	2023	Unclear risk	Low risk	Unclear risk	Unclear risk	Unclear risk	Low risk	Low risk	Low risk	Low risk	Low risk
Li et.al[13]	2020	Unclear risk	Low risk	Unclear risk	Unclear risk	Unclear risk	Low risk	Unclear risk	Low risk	Low risk	Low risk
Li et.al[14]	2022	Unclear risk	Low risk	Unclear risk	Unclear risk	Unclear risk	Low risk	Unclear risk	Low risk	Low risk	Low risk
Li et.al[15]	2022	Unclear risk	Low risk	Unclear risk	Low risk	Unclear risk	Low risk	Unclear risk	Low risk	Low risk	Low risk
Liu et.al[16]	2021	Unclear risk	Low risk	Unclear risk	Unclear risk	Unclear risk	Low risk	Unclear risk	Low risk	Low risk	Low risk
Liu et.al[17]	2021	Unclear risk	Low risk	Unclear risk	Unclear risk	Unclear risk	Low risk	Unclear risk	Low risk	Low risk	Low risk
Ren	2021	Unclea	Low	Unclea	Unclear	Unclear	Low risk	Unclear risk	Low risk	Low risk	Low risk

et.al[18]		r risk	risk	r risk	risk	risk					
Shen et.al[19]	2019	Unclear risk	Low risk	Unclear risk	Low risk	Unclear risk	Low risk	Unclear risk	Low risk	Low risk	Low risk
Shi et.al[20]	2020	Unclear risk	Low risk	Unclear risk	Unclear risk	Unclear risk	Low risk	Unclear risk	Low risk	Low risk	Low risk
Shi et.al[21]	2020	Unclear risk	Low risk	Unclear risk	Unclear risk	Unclear risk	Low risk	Unclear risk	Low risk	Low risk	Low risk
Song et.al[22]	2022	Unclear risk	Low risk	Unclear risk	Unclear risk	Unclear risk	Low risk	Unclear risk	Low risk	Low risk	Low risk
Tao et.al[23]	2021	Unclear risk	Low risk	Unclear risk	Low risk	Unclear risk	Low risk	Low risk	Low risk	Low risk	Low risk
Wang et.al[24]	2019	Unclear risk	Low risk	Unclear risk	Unclear risk	Unclear risk	Low risk	Unclear risk	Low risk	Low risk	Low risk
Wang et.al[25]	2019	Unclear risk	Low risk	Unclear risk	Low risk	Unclear risk	Low risk	Unclear risk	Low risk	Low risk	Low risk
Wang et.al[26]	2022	Unclear risk	Low risk	Unclear risk	Unclear risk	Unclear risk	Low risk	Low risk	Low risk	Low risk	Low risk
Wang et.al[27]	2021	Unclear risk	Low risk	Unclear risk	Unclear risk	Unclear risk	Low risk	Low risk	Low risk	Low risk	Low risk
Wang et.al[28]	2021	Unclear risk	Low risk	Unclear risk	Low risk	Unclear risk	Low risk	Unclear risk	Low risk	Low risk	Low risk
Wang et.al[29]	2020	Unclear risk	Low risk	Unclear risk	Unclear risk	Unclear risk	Low risk	Low risk	Low risk	Low risk	Low risk
Wellings et.al[30]	2021	Unclear risk	Low risk	Unclear risk	High risk	High risk	Low risk	Unclear risk	Low risk	Low risk	Low risk
Wu et.al[31]	2022	Unclear risk	Low risk	Unclear risk	Unclear risk	Unclear risk	Low risk	Unclear risk	Low risk	Low risk	Low risk
Wu et.al[32]	2021	Unclear risk	Low risk	Unclear risk	Unclear risk	Unclear risk	Low risk	Unclear risk	Low risk	Low risk	Low risk
Xu et.al[33]	2022	Unclear risk	Low risk	Unclear risk	Unclear risk	Unclear risk	Low risk	Low risk	Low risk	Low risk	Low risk
Xu et.al[34]	2023	Unclear risk	Low risk	Unclear risk	Unclear risk	Unclear risk	Low risk	Low risk	Low risk	Low risk	Low risk
Xu et.al[35]	2022	Unclear risk	Low risk	Unclear risk	Unclear risk	Unclear risk	Low risk	Low risk	Low risk	Low risk	Low risk
Yao et.al[36]	2020	Unclear risk	Low risk	Unclear risk	Low risk	Unclear risk	Low risk	Unclear risk	Low risk	Low risk	Low risk
Yao et.al[37]	2021	Unclear risk	Low risk	Unclear risk	Unclear risk	Unclear risk	Low risk	Unclear risk	Low risk	Low risk	Low risk
Ye et.al[38]	2023	Unclear risk	Low risk	Unclear risk	Low risk	Unclear risk	Low risk	Unclear risk	Low risk	Low risk	Low risk
Yu et.al[39]	2021	Unclear risk	Low risk	Unclear risk	Unclear risk	Unclear risk	Low risk	Unclear risk	Low risk	Low risk	Low risk
Yu et.al[40]	2020	Unclear risk	Low risk	Unclear risk	Unclear risk	Unclear risk	Low risk	Low risk	Low risk	Low risk	Low risk
Zhang et.al[41]	2022	Unclear risk	Low risk	Unclear risk	Unclear risk	Unclear risk	Low risk	Low risk	Low risk	Low risk	Low risk

Zhang et.al[42]	2022	Unclear risk	Low risk	Unclear risk	Unclear risk	Unclear risk	Low risk	Unclear risk	Low risk	Low risk	Low risk
Zhang et.al[43]	2020	Unclear risk	Low risk	Unclear risk	Unclear risk	Unclear risk	Low risk	Unclear risk	Low risk	Low risk	Low risk
Zhang et.al[44]	2022	Unclear risk	Low risk	Unclear risk	Unclear risk	Unclear risk	Low risk	Low risk	Low risk	Low risk	Low risk
Zhao et.al[45]	2022	Unclear risk	Low risk	Unclear risk	Unclear risk	Unclear risk	Low risk	Unclear risk	Low risk	Low risk	Low risk
Zhu et.al[46]	2022	Unclear risk	Low risk	Unclear risk	Low risk	Unclear risk	Low risk	Low risk	Low risk	Low risk	Low risk

Table S5: Summary of parameters of exosomes (Tendon healing and tendon-bone healing)

Author	Year	Origin/Source	Isolation method	Storage	Characterization	Size Distribution	EV/Exosome makers
Chamberlain et.al[1]	2019	Human BM-MSC exosomes	Differential centrifugation and ultracentrifugation	NR	NTA, FCM	61 ~ 121 nm	CD146, CD29, CD44, CD63, CD81, CD105
Chen et.al[2]	2021	Rabbit ASC exosomes	Differential centrifugation	-80°C	NTA, TEM, WB	99.4~233.4 nm	CD9, CD63
Cui et.al[3]	2019	Mouse BMDM exosomes	Centrifugation and ultracentrifugation	NR	NTA, TEM, WB	70~150 nm	CD9, CD63, TSG101, Alix
Davies et.al[4]	2022	Mouse FAP	Differential centrifugation and ultracentrifugation	4°C	NTA	NR	NR
Fu et.al[5]	2021	Human ASC exosomes	Differential centrifugation and ultracentrifugation	NR	NTA, TEM, WB	30 ~ 150 nm	CD9, HSP70, TSG101 +ve; Calnexin -ve
Gao et.al[6]	2022	Human IP-MSC exosomes	Differential centrifugation and ultracentrifugation	NR	NFA, TEM, WB	50 ~ 200 nm	CD9, CD63, TSG101 +ve; GM130 -ve
Gissi et.al[7]	2020	Rat BM-MSC exosomes	Differential centrifugation and ultracentrifugation	-80°C	AFM, CONAN, WB	30~200nm	Annexin XI, Annexin V, TSG101, CD29, TERT
Han et.al[8]	2022	HU-MSC exosomes	Differential centrifugation and ultracentrifugation	NR	TEM, Nano FCM software, WB	42~197 nm	CD9, TSG101, Alix +ve; Calnexin -ve
Han et.al[9]	2022	Rabbit BM-MSC exosomes	Exosomes were extracted by Total Exosome Isolation Reagent	NR	NTA, TEM, WB, FCM	100 ~ 200 nm	CD9, CD63, ALIX, Annexin V, TSG101 +ve; Calnexin -ve
Hayashi et.al[10]	2022	Human BM-MSC exosomes	Differential centrifugation and ultracentrifugation	NR	NTA, WB	P5 EVs: 148±7 nm<br>P12 EVs: 158±10 nm	CD63, CD81, Flotilin-1
Huang et.al[11]	2020	Rat BM-MSC exosomes	Differential centrifugation and	-80°C	TEM, WB	30 ~ 150 nm	CD9, CD63, CD 81

			ultracentrifugation				
Jenner et.al[12]	2023	HU-MSC exosomes	Ultracentrifugation	-80°C	NTA, TEM, FCM	~126.63 nm	CD9, CD29, CD63, CD81
Li et.al[14]	2022	Rat BM-MSC exosomes	Differential centrifugation and ultracentrifugation	NR	NTA, TEM, WB	30 ~ 200 nm	CD9, HSP70, TSG101
Li et.al[15]	2022	Rat BM-MSC exosomes	Differential centrifugation and ultracentrifugation	NR	NTA, TEM, WB	~ 100 nm	CD9, CD63, TSG101 +ve; Calnexin -ve
Li et.al[13]	2020	HU-MSC exosomes	Differential centrifugation and ultracentrifugation	NR	NTA, TEM, WB	30 ~ 150 nm	CD9, CD63, TSG101, Alix +ve; Calnexin -ve
Liu et.al[16]	2021	Rat ASC exosomes	Differential centrifugation and ultracentrifugation	NR	NTA, TEM, WB	~ 109.6 nm	CD9, TSG101, HSP70
Liu et.al[17]	2021	Rat TSC	Differential centrifugation and ultracentrifugation	NR	NTA, TEM, WB	100~200 nm	CD9, CD63, TSG101
Ren et.al[18]	2021	PEP	NA	-80°C	NTA, TEM	56.4~151 nm	NA
Shen et.al[19]	2019	Mouse ASC exosomes	Differential centrifugation and ultracentrifugation	NR	TEM, NTA, WB	IEVs: 108±2 nm EVs: 113±3 nm	CD9, CD63
Shi et.al[20]	2020	Mouse BM-MSC exosomes	Differential centrifugation and ultracentrifugation	-80°C	NTA, TEM, WB	~120.3 nm	CD9, CD81, TSG101
Shi et.al[21]	2020	PEP	NA	NR	NTA, TEM	93.70~124.65 nm	NA
Song et.al[22]	2022	Rat TSC exosomes	Differential centrifugation and ultracentrifugation	NR	NTA, TEM, WB	~139 nm	CD9, CD63, CD81, TSG101
Tao et.al[23]	2021	Human TSPC exosomes	Differential centrifugation and ultracentrifugation	NR	TEM, DLS, WB	73.59±38.15 nm	CD9, CD63, CD81
Wang et.al[26]	2022	Human ASC exosomes	Differential centrifugation and ultracentrifugation	NR	NR	NR	NR
Wang et.al[27]	2021	Human ASC exosomes	Differential centrifugation and ultracentrifugation	NR	NR	NR	NR
Wang et.al[28]	2021	Mouse BM-MSC exosomes	Differential centrifugation and ultracentrifugation	NR	NTA, TEM, WB	100~150 nm	Hsp70, CD9, CD63, CD81, TSG101
Wang et.al[29]	2020	Human ASC exosomes	Differential centrifugation and ultracentrifugation	NR	TEM, NTA, WB	50~150 nm	CD9, CD63, TSG101 +ve; GM130 -ve
Wang et.al[24]	2019	Human ASC exosomes	Differential centrifugation and ultracentrifugation	NR	TEM, NTA, WB	50~150 nm	CD9, CD63, TSG101 +ve; GM130 -ve

Wang et.al[25]	2019	Rat TSC exosomes	Differential centrifugation and ultracentrifugation	NR	TEM, WB	40~200 nm	CD63, CD81
Wellings et.al[30]	2021	PEP	NA	NR	NR	NR	NA
Wu et.al[31]	2022	Human BM-MSC exosomes	Differential centrifugation and ultracentrifugation	-80°C	NTA, TEM, WB	80 ~ 160 nm	Hsp70, CD9, CD81, TSG 101 +ve; Calnexin -ve
Wu et.al[32]	2021	Rat BM-MSC exosomes	Differential centrifugation and ultracentrifugation	-80°C	NTA, TEM, WB	50~200 nm	CD9, CD63, TSG101 +ve; Calnexin -ve
Xu et.al[33]	2022	Rat ASC	Differential centrifugation and ultracentrifugation	-80°C	NTA, TEM, WB	Exosomes: 145.96±0.5 nm Ectosomes: 227.66±4.9 nm	CD9、CD63、TSG101、Hsp70、Alix +ve；Calnexin -ve
Xu et.al[34]	2023	Human ASC exosomes	Differential centrifugation	-80°C	NTA, TEM, WB	EV_N: 155.9±27.6 nm EV_B: 146.8±15.2 nm	CD9, CD63 +ve; Calnexin -ve
Xu et.al[35]	2022	Rat IPFP-MSC exosomes	Differential centrifugation and ultracentrifugation	NR	NTA, TEM, WB	50 ~ 150 nm	CD63, CD81, TSG101
Yao et.al[36]	2020	HU-MSC exosomes	Differential centrifugation and ultracentrifugation	-80°C	NTA, TEM, WB	30 ~ 200 nm	CD9, CD63, Alix, TSG101
Yao et.al[37]	2021	HU-MSC exosomes	Differential centrifugation and ultracentrifugation	-80°C	NTA, TEM, WB	~137 nm	CD9, CD63, Alix
Ye et.al[38]	2023	Human IP-MSC exosomes	Differential centrifugation and ultracentrifugation	-80°C	NTA, TEM, WB	Small EVs: ~100 nm Large EVs: ~225 nm	CD9, CD63, TSG101 +ve; GM130 -ve
Yu et.al[39]	2021	Mouse BMDM exosomes	Centrifugation and ultracentrifugation	NR	TEM, WB	70~150 nm	CD63, CD81, TSG101
Yu et.al[40]	2020	Rat BM-MSC exosomes	Differential centrifugation and ultracentrifugation	-80°C	NTA, TEM, WB	50.5~151.7 nm	CD9, TSG101, Alix
Zhang et.al[41]	2022	Rabbit BM-MSC exosomes	Differential centrifugation and ultracentrifugation	NR	NTA, TEM, WB	50 ~ 150 nm	CD9, CD63, TSG101, Alix
Zhang et.al[42]	2022	Rat ASC exosomes	Differential centrifugation and ultracentrifugation	NR	NTA, TEM, WB	50~150 nm	CD9, CD63, TSG101
Zhang et.al[43]	2020	Rat TSC exosomes	Differential centrifugation and ultracentrifugation	NR	TEM, WB	~116 nm	CD9, TSG101, HSP70
Zhang et.al[44]	2022	Rat BM-MSC	Differential	NR	NTA, TEM, WB	50~100 nm	CD63, CD81, TSG101

		exosomes	centrifugation and ultracentrifugation				
Zhao et.al[45]	2022	Rat ASC exosomes	Differential centrifugation and ultracentrifugation	NR	TEM, DLS, WB	100~200 nm	CD9, CD63, TSG101
Zhu et.al[46]	2022	Human IP-MSC exosomes	Differential centrifugation and ultracentrifugation	-80°C	NFA, TEM, WB	60~160 nm	CD9、TSG101、CD63 +ve；GM130 -ve

IP-MSC, induced pluripotent mesenchymal stem cells; NFA, Nano-flow analysis; TEM, Transmission electron microscope; WB, Western blot; BM-MSC, bone marrow mesenchymal stem cell; NR, not reported; P5 EVs, extracellular vesicles from mesenchymal stem cell at passage 5; P12 EVs, extracellular vesicles from mesenchymal stem cell at passage 12; NTA, nanoparticle tracking analysis; TSC, tendon stem cell; ASC, Adipose stem cell; IEVs, IFN γ-primed Extracellular vesicles; EVs, Extracellular vesicles; TSPC, tendon stem/progenitor cell; DLS, Dynamic Light Scattering; PEP, Purified Exosome Product; NA, not available; FAP, Fibroadipogenic progenitor; HU-MSC, Human umbilical cord mesenchymal stem cell; BMDMs, bone marrow-derived macrophages; AFM Atomic Force Microscopy; CONAN Colloidal Nano plasmonic assay; FCM, Flow Cytometry; EV_B, bioactive glasses-elicited mesenchymal stem cell extracellular vesicle; EV_N, native mesenchymal stem cell extracellular vesicle; IPFP MSC, Infrapatellar fat pad mesenchymal stem cell

Table S6: Summary of animal studies and treatment parameters. (Tendon healing)

Author	Year	Group/treatment	Concentration	Volume	Delivery route	Frequency	Euthanasia
Chamberlain et.al[1]	2019	(1) No treatment	NA	NA	NA	NA	1, 2 weeks
		(2) MSC	NR	NR	Local Injection	At day 0	1, 2 weeks
		(3) MQ	NR	NR	Local Injection	At day 0	1, 2 weeks
		(4) EEM	NR	NR	Local Injection	At day 0	1, 2 weeks
		(5) Exosomes	NR	NR	Local Injection	At day 0	1, 2 weeks
Chen et.al[2]	2021	(1) PBS	NA	NR	Local Injection	At day 0	4 weeks
		(2) Exosomes	NR	NR	Local Injection	At day 0	4 weeks
Cui et.al[3]	2019	(1) BDMD exosomes	NR	20 mg	Intravenous injection	Once every 3 days	3 weeks
		(2) Empty liposomes	NA	20 mg	Intravenous injection	Once every 3 days	3 weeks
Davies et.al[4]	2022	(1) PBS	NA	NR	Local Injection	At day 0	6 weeks
		(2) Exosomes from UCP1++ cells	2×10^9 particles	NR	Local Injection	At day 0	6 weeks
		(3) Exosomes from UCP1- cells	2×10^9 particles	NR	Local Injection	At day 0	6 weeks
Fu et.al[5]	2021	(1) PBS	NA	100 μL	Local Injection	At week 4	8, 12 weeks
		(2) Hydrogel	NA	100 μL	Local Injection	At week 4	8, 12 weeks
		(3) Exosomes with hydrogel	0.3 mg/mL	100 μL	Local Injection	At week 4	8, 12 weeks
		(4) No treatment	NA	NA	NA	NA	8, 12 weeks
Gao et.al[6]	2022	(1) Sham group	NA	NA	NA	NA	5 weeks
		(2) PBS	NA	NR	Local Injection	At week 1	5 weeks
		(3) Exosomes	1×10^9 particles/ml	NR	Local Injection	At week 1	5 weeks

Gissi et.al[7]	2020	(1) PBS	NA	50 µL	Local Injection	At day 0	30 days
		(2) Rat BM-MSC with PBS	4×10^{6} particles/50 µL	50 µL	Local Injection	At day 0	30 days
		(3) EV_L with PBS	2.8×10^{12} particles/mL	50 µL	Local Injection	At day 0	30 days
		(4) EV_H with PBS	8.4×10^{12} particles/mL	50 µL	Local Injection	At day 0	30 days
Han et.al[8]	2022	(1) No treatment	NA	NA	NA	NA	1 week
		(2) Sham group	NA	NA	NA	NA	1 week
		(3) Exosomes	100 µg/50 µL	50 µL	Subcutaneous injection	At day 0	1 week
Hayashi et.al[10]	2022	(1) P5 EVs with PBS	3595±480 ng/20 µL	20 µL	Local Injection	At day 1 and day 7	1, 4 weeks
		(2) P12 EVs with PBS	3655±626 ng/20 µL	20 µL	Local Injection	At day 1 and day 7	1, 4 weeks
		(3) PBS	NA	20 µL	Local Injection	At day 1 and day 7	1, 4 weeks
Jenner et.al[12]	2023	(1) Ringer lactate solution and collagen sponge	NA	100 µL	Implantation	At day 0	6 weeks
		(2) Exosomes with ringer lactate solution and collagen sponge	2×10^{10} particles in 100 µL	100 µL	Implantation	At day 0	6 weeks
Li et.al[14]	2022	(1) Fibrin sealant	NA	30 µL	Local Injection	At day 0	1, 2, 8 weeks
		(2) TSCs with fibrin sealant	5×10^{4} particles in 30 µL	30 µL	Local Injection	At day 0	1, 2, 8 weeks
		(3) BM-MSC-EV treated TSCs with fibrin sealant	5×10^{4} particles in 30 µL	30 µL	Local Injection	At day 0	1, 2, 8 weeks
		(4) EUG-BM-MSC-EV treated TSCs with fibrin sealant	5×10^{4} particles in 30 µL	30 µL	Local Injection	At day 0	1, 2, 8 weeks
Li et.al[13]	2020	(1) PBS	NA	50 µL	Subcutaneous injection	At day 0	3 weeks
		(2) Exosomes	200 µg/50 µL	50 µL	Subcutaneous injection	At day 0	3 weeks
		(3) HCPT-exosomes	200 µg/50 µL	50 µL	Subcutaneous injection	At day 0	3 weeks
Liu et.al[16]	2021	(1) Sham group	NA	NA	NA	NA	1, 2, 4 weeks
		(2) GelMA	NA	30 µL	Implantation	At day 0	1, 2, 4 weeks
		(3) Exosomes with GelMA	200 µg/30 µL	30 µL	Implantation	At day 0	1, 2, 4 weeks

Liu et.al[17]	2021	(1)	No treatment	NA	NA	NA	NA	4 weeks
		(2)	Sham group	NA	NA	NA	NA	4 weeks
		(3)	Exosomes	NR	NR	Local Injection	At week 2	4 weeks
		(4)	Exosomes/MBA	NR	NR	Transdermal delivery	At week 2	4 weeks
Shen et.al[19]	2019	(1)	Collagen sheet	NA	5 mm^3	Implantation	At day 0	7 days
		(2)	Collagen sheet loaded with EVs	5~6× 10^9 particles/sheet	5 mm^3	Implantation	At day 0	7 days
		(3)	Collagen sheet loaded with IEVs	5~6× 10^9 particles/sheet	5 mm^3	Implantation	At day 0	7 days
Shi et.al[21]	2020	(1)	No treatment	NA	NA	NA	NA	4 weeks
		(2)	Tisseel patch	NA	NR	Implantation	At day 0	4 weeks
		(3)	PEP with Tisseel patch	1× 10^{12} particles/ml	NR	Implantation	At day 0	4 weeks
Song et.al[22]	2022	(1)	Sham group	NA	NA	NA	NA	2, 4, 8 weeks
		(2)	PHA	NA	200μL	Local Injection	At day 0	2, 4, 8 weeks
		(3)	Exosomes with PHA	2 × 10^{10} particles/200 μL	200 μL	Local Injection	At day 0	2, 4, 8 weeks
Tao et.al[23]	2021	(1)	Sham group	NA	NA	NA	NA	4 weeks
		(2)	Hydrogel	1× 10^{11} particles/ml	0.1 cm^3	Implantation	At day 0	4 weeks
		(3)	Con-EVs with hydrogel	1× 10^{11} particles/ml	0.1 cm^3	Implantation	At day 0	4 weeks
		(4)	H19-sOE-EVs with hydrogel	1× 10^{11} particles/ml	0.1 cm^3	Implantation	At day 0	4 weeks
		(5)	hnRNP A2/B1-EVs with hydrogel	1× 10^{11} particles/ml	0.1 cm^3	Implantation	At day 0	4 weeks
		(6)	H19-OL-EVs with hydrogel	1× 10^{11} particles/ml	0.1 cm^3	Implantation	At day 0	4 weeks
Wang et.al[26]	2022	(1)	No treatment	NA	NA	NA	NA	6, 12 weeks
		(2)	Fibrin	NA	NR	Local Injection	At day 0	6, 12 weeks
		(3)	Exosomes with fibrin	1× 10^{11} particles/mL	NR	Local Injection	At day 0	6, 12 weeks
		(4)	Sham group	NA	NA	NA	NA	12 weeks
Wang et.al[27]	2021	(1)	Saline	NA	10 μL	Injection around the enthesis site	At week 6	10 weeks
		(2)	Exosomes with saline	1× 10^{11}particles in 10 μL	10 μL	Injection around the enthesis site	At week 6	10 weeks
		(3)	Saline	NA	10 μL	Injection around the enthesis site	At week 6	10 weeks

Wang et.al[24]	2019	(1)	Sham group	NA	NA	NA	NA	8, 16 weeks
		(2)	Saline	NA	20 μL	Local Injection	At day 0	8, 16 weeks
		(3)	Exosomes with saline	1×10^{11} particles/20 μL	20 μL	Local Injection	At day 0	8, 16 weeks
Wang et.al[25]		(1)	No treatment	NA	NA	NA	NA	5 weeks
	2019	(2)	Rat TSCs	1×10^{6} particles in 20 μL	20 μL	Local Injection	Twice a week	5 weeks
		(3)	Exosomes from rat TSCs	0.486 μg/μL	20 μL	Local Injection	Twice a week	5 weeks
Wellings et.al[30]	2021	(1)	Sham group	NA	NA	NA	NA	3, 6 weeks
		(2)	Type 1 collagen scaffold	NA	NR	Apply at the repair site	At day 0	3, 6 weeks
		(3)	PEP with type 1 collagen scaffold	1×10^{11} particles/ml	NR	Apply at the repair site	At day 0	3, 6 weeks
Xu et.al[33]		(1)	Saline	NA	25 μL	Local Injection	At week 1	3, 5 weeks
	2022	(2)	Exosomes	1×10^{10} particles in 25 μL	25 μL	Local Injection	At week 1	3, 5 weeks
		(3)	Ectosomes	1×10^{10} particles in 25 μL	25 μL	Local Injection	At week 1	3, 5 weeks
Xu et.al[34]		(1)	Intact, no treatment	NA	NA	NA	NA	3 days, 1, 2, 4 weeks
		(2)	Surgery, no treatment	NA	NA	NA	NA	3 days, 1, 2, 4 weeks
	2023	(3)	GelMA hydrogel	NA	20 μL	Implantation	At day 0	3 days, 1, 2, 4 weeks
		(4)	EV_B with GelMA hydrogel	80 μg/20 μL	20 μL	Implantation	At day 0	3 days, 1, 2, 4 weeks
		(5)	EV_N with GelMA hydrogel	80 μg/20 μL	20 μL	Implantation	At day 0	3 days, 1, 2, 4 weeks
Yao et.al[36]	2020	(1)	Sham group	NA	NA	NA	NA	3 weeks
		(2)	Exosomes with PBS	200 μg/50 μL	50 μL	Subcutaneous injection	At day 0	3 weeks
		(3)	PBS	NA	50 μL	Subcutaneous injection	At day 0	3 weeks
Yao et.al[37]	2021	(1)	Sham group	NA	NA	NA	NA	2, 4 weeks
		(2)	Fibrin glue	NA	50 μL	Implantation	At day 0	2, 4 weeks
		(3)	Fibrin glue with HU-MSC exosomes	100 μg/50 μL	50 μL	Implantation	At day 0	2, 4 weeks
		(4)	Sham group	NA	NA	NA	NA	4 weeks
		(5)	Fibrin glue with agomir-NC exosomes	100 μg/50 μL	50 μL, 100 μg	Implantation	At day 0	4 weeks
		(6)	Fibrin glue with agomir-29a exosomes	100 μg/50 μL	50 μL, 100 μg	Implantation	At day 0	4 weeks

Ye et.al[38]	2023	(1) Sham group	NA	NA	NA	NA	2, 4 weeks
		(2) PBS	NA	100 µL	Local injection	At day 0	2, 4 weeks
		(3) Small EVs	1× 10^{10} particles/ml	100 µL	Local injection	At day 0	2, 4 weeks
		(4) Large EVs	1× 10^{9} particles/ml	100 µL	Local injection	At day 0	2, 4 weeks
Yu et.al[39]	2021	(1) No treatment	NA	NA	NA	NA	2, 3 weeks
		(2) Sham group	NA	NA	NA	NA	2, 3 weeks
		(3) M2 macrophage exosomes	NR	NR	NR	At day 0	2, 3 weeks
		(4) CircRNA-Ep-400 depletion of M2 macrophage exosomes	NR	NR	NR	At day 0	2, 3 weeks
Yu et.al[40]	2020	(1)Exosomes with fibrin	2 µg/µL	10 µL	Local Injection	At day 0	3 days, 1, 2, 4 weeks
		(2) PBS with fibrin	NA	10 µL	Local Injection	At day 0	3 days, 1, 2, 4 weeks
Zhang et.al[41]	2022	(1) Sham group	NA	NA	NA	NA	16, 20, 24 weeks
		(2) Fiber-aligned patch	NA	$1cm^2$ (size of the patch)	Fix the patch on supraspinatus	At week 12	16, 20, 24 weeks
		(3) Exosomes-loaded fiber-aligned patch	NR	$1cm^2$ (size of the patch)	Fix the patch on supraspinatus	At week 12	16, 20, 24 weeks
Zhang et.al[42]	2022	(1) Saline	0.1 mg/kg	NR	Local Injection	At week 6	7 weeks
		(2) Dexamethasone	0.1 mg/kg	NR	Local Injection	At week 6	7 weeks
		(3) Exosomes with dexamethasone	1× 10^{11} Particles/mL	NR	Local Injection	At week 6	7 weeks
Zhang et.al[43]	2020	(1) GelMA with Exosomes from TSCs	200 µg/30 µL	30 µL	Local Injection	At day 0	1, 2, 8 weeks
		(2) GelMA group	NA	30 µL	Local Injection	At day 0	1, 2, 8 weeks
		(3) No treatment	NA	NA	NA	NA	1, 2, 8 weeks
Zhao et.al[45]	2022	(1) Saline	NA	NR	Local Injection	At week 1	5 weeks
		(2) ASC with saline	1× 10^{6} particles	NR	Local Injection	At week 1	5 weeks
		(3) Exosomes with saline	50 µg	NR	Local Injection	At week 1	5 weeks
Zhu et.al[46]	2022	(1) Sham tendinopathy group	NA	NA	NA	NA	8 weeks
		(2) PBS group	NA	100 µL	Local Injection	weekly	8 weeks
		(3) PBS with Exosomes from IP-MSC	1× 10^{10} particles in 100 µL	100 µL	Local Injection	Weekly	8 weeks
		(4)					
		(5) Steroid-treated group	NA	100 µL	Local Injection	weekly	8 weeks

PBS, phosphate-buffered saline; IP-MSC, induced pluripotent mesenchymal stem cells; NA, not available; P5 EVs, extracellular vesicles from

mesenchymal stem cell at passage 5; P12 EVs, extracellular vesicles from mesenchymal stem cell at passage 12; GelMA, gelatin methacryloyl; TSCs, tendon stem cells; EVs, Extracellular vesicles; IEVs, IFN γ-primed Extracellular vesicles; Con-EVs, control extracellular vesicles; H19-sOE-EVs, extracellular vesicles derived from tendon stem/progenitor cells over-expressing H19; hnRNP A2/B1-EVs, extracellular vesicles derived from tendon stem/progenitor cells over-expressing hnRNP A2/B1; H19-OL-EVs, extracellular vesicles derived from tendon stem/progenitor cells co-overexpressed of H19 and hnRNP A2/B1; NR, not reported; PEP, purified exosome product; Exosomes/MBA, exosomes modified by a nitric oxide nanomotor; UCP1++ cells, Fibroadipogenic progenitors exhibiting a high UCP1 fluorescent intensity > 10^4, UCP1- cells, Fibroadipogenic progenitors that were UCP1-negative were defined as TdTomato fluorescent intensity < 10^2; HU-MSC, Human umbilical cord mesenchymal stem cell; BMDM, bone marrow-derived macrophages; BM-MSCs, bone morrow mesenchymal stem cells; EV_L, low concentration of Extracellular vesicles; EV_H, high concentration of Extracellular vesicles; HCPT-exosomes, exosomes derived from human umbilical cord mesenchymal stem cell treated with hydroxycamptothecin; MSC, mesenchymal stem cells; MQ, macrophage; EEM, exosome-educated macrophages; PHA, photopolymerizable hyaluronic acid; BM-MSC-EV, bone morrow mesenchymal stem cells extracellular vesicle; EUG-BM-MSC-EV, eugenol treated-bone morrow mesenchymal stem cells extracellular vesicle; EV_B, bioactive glasses-elicited mesenchymal stem cell extracellular vesicle; EV_N, native mesenchymal stem cell extracellular vesicle; ASC, Adipose stem cell

Table S7: Summary of animal studies and treatment parameters (Tendon-bone healing)

Author	Year	Group/treatment	Concentration	Volume	Delivery route	Frequency	Euthanasia
Han et.al[9]	2022	(1) Sham group	NA	NA	NA	NA	12 weeks
		(2) Exosomes	NR	NR	Injection into the key bone interface	At day 0	12 weeks
		(3) Exosomes with empty microcapsules	NR	NR	Injection into the key bone interface	At day 0	12 weeks
		(4) Exosomes with BMP-2-loaded microcapsules	NR	NR	Injection into the key bone interface	At day 0	12 weeks
Huang et.al[11]	2020	(1) PBS	NA	200 μL	Injection into the tail vein	At week 4	8, 12 weeks
		(2) Exosomes with PBS	200 μg/200 μL	200 μL	Injection into the tail vein	At week 4	8, 12 weeks
Li et.al[15]	2022	(1) PBS	NA	50 μL	Injection into the joint cavity	At day 0, day 3, day 7	4, 8 weeks
		(2) BM-MSC Exosomes	1×10^{11} Particles/mL	50 μL	Injection into the joint cavity	At day 0, day 3, day 7	4, 8 weeks
		(3) miR-23a-3p overexpression BM-MSC exosomes	1×10^{11} Particles/mL	50 μL	Injection into the joint cavity	At day 0, day 3, day 7	4, 8 weeks
Ren et.al[18]	2021	(1) No treatment	NA	NA	NA	NA	6 weeks
		(2) Fibrin sealant	NA	$27mm^3$	Implantation	At day 0	6 weeks
		(3) Exosomes with fibrin sealant	20% (vol/vol)	$27mm^3$	Implantation	At day 0	6 weeks
Shi et.al[20]	2020	(1) Control group	NA	NR	Local Injection	At day 0	7, 14, 30 days
		(2) Hydrogel	NR	NR	Local Injection	At day 0	7, 14, 30 days
		(3) Exosomes with hydrogel	NR	NR	Local Injection	At day 0	7, 14, 30 days

Wang et.al[28]	2021	(1)	Control group	NA	NR	Injected into bone tunnels	At day 0	NR
		(2)	BM-MSC exosomes	1×10^{10} Particles/leg	NR	Injected into bone tunnels	At day 0	NR
		(3)	$BM-MSC^{AD}$ exosomes	1×10^{10} Particles/leg	NR	Injected into bone tunnels	At day 0	NR
		(4)	$BM-MSC^{SCX}$ exosomes	1×10^{10} Particles/leg	NR	Injected into bone tunnels	At day 0	NR
Wang et.al[29]	2020	(1)	Sham group 1	NA	NA	NA	NA	6 weeks
		(2)	Sham group 2	NA	NA	NA	NA	18 weeks
		(3)	No treatment	NA	NA	NA	NA	6 weeks
		(4)	Saline	NA	20μL/per side	Local Injection	At week 6	18 weeks
		(5)	Exosomes with saline	1×10^{11} Particles/20 μL	20μL/per side	Local Injection	At week 6	18 weeks
Wu et.al[31]	2022	(1)	PBS	NA	NR	Local injection	At day 0	2, 4, 8 weeks
		(2)	Exosomes	100μg/tunnel	NR	Local injection	At day 0	2, 4, 8 weeks
		(3)	IONP-exosomes	100μg/tunnel	NR	Local injection	At day 0	2, 4, 8 weeks
Wu et.al[32]	2021	(1)	Saline	NA	20 μL	Local Injection	At day 0	2, 4 weeks
		(2)	BM-MSC exosomes	1×10^{11} Particles/20μL	20 μL	Local Injection	At day 0	2, 4 weeks
		(3)	LIPUS-BM-MSC exosomes	1×10^{11} Particles/20μL	20 μL	Local Injection	At day 0	2, 4 weeks
Xu et.al[35]	2022	(1)	No treatment	NA	NA	NA	NA	2, 4, 8 weeks
		(2)	PBS and SAH	NA	Intra-tunnel: 0.1 mL;Intra-articular: 0.2 mL	Intra-tunnel injection (SAH) and intra-articular injection (PBS)	Intra-tunnel injection: at day 0 intra-articular injection: weekly	2, 4, 8 weeks
		(3)	IME and IME-loaded SAH	1×10^{10} Particles/mL	Intra-tunnel: 0.1 mL;Intra-articular: 0.2 mL	Intra-tunnel injection (IME-loaded SAH) and intra-articular injection (IME)	Intra-tunnel injection: at day 0 intra-articular injection: weekly	2, 4, 8 weeks

Zhang et.al[44]		(1)	PBS	NA	300 μL	Injection into bone tunnels at 12, 4, and 8 o'clock directions	At day 0	2, 4, 8 weeks
	2022	(2)	Normal exosomes with adhesive hydrogel	10 mg/mL	300 μL	Injection into bone tunnels at 12, 4, and 8 o'clock directions	At day 0	2, 4, 8 weeks
		(3)	Hypo-exosomes with adhesive hydrogel	10 mg/mL	300 μL	Injection into bone tunnels at 12, 4, and 8 o'clock directions	At day 0	2, 4, 8 weeks

PBS, phosphate-buffered saline; NA, not available; NR, not reported; BM-MSC, bone morrow mesenchymal stem cell; $\mathrm{BM-MSC^{SCX}}$, Scleraxis-overexpressing bone morrow mesenchymal stem cell; $\mathrm{BM-MSC^{AD}}$, bone morrow mesenchymal stem cell transfected with empty vectors; SAH, sodium alginate hydrogel; IME, infrapatellar fat pad mesenchymal stem cell-derived exosome; Hypo-exosomes, exosomes secreted by hypoxia-stimulated bone-marrow mesenchymal stem cells; IONP-exosomes, Exosomes derived from magnetically actuated bone morrow mesenchymal stem cells; BMP, Bone morphogenetic protein; LIPUS-BM-MSC, Bone Marrow Mesenchymal Stem Cell Preconditioned by Low-Intensity Pulsed Ultrasound Stimulation;

Reference

1. Chamberlain CS, Clements AEB, Kink JA, et al. Extracellular Vesicle-Educated Macrophages Promote Early Achilles Tendon Healing. Article. *Stem Cells*. 2019;37(5):652-662.
2. Chen SH, Chen ZY, Lin YH, et al. Extracellular vesicles of adipose-derived stem cells promote the healing of traumatized achilles tendons. Article. *International Journal of Molecular Sciences*. 2021;22(22)
3. Cui H, He Y, Chen S, Zhang D, Yu Y, Fan C. Macrophage-Derived miRNA-Containing Exosomes Induce Peritendinous Fibrosis after Tendon Injury through the miR-21-5p/Smad7 Pathway. Article. *Molecular Therapy - Nucleic Acids*. 2019;14:114-130.
4. Davies MR, Garcia S, Liu M, et al. Muscle-Derived Beige Adipose Precursors Secrete Promyogenic Exosomes That Treat Rotator Cuff Muscle Degeneration in Mice and Are Identified in Humans by Single-Cell RNA Sequencing. Article. *The American journal of sports medicine*. 2022;50(8):2247-2257.
5. Fu G, Lu L, Pan Z, Fan A, Yin F. Adipose-derived stem cell exosomes facilitate rotator cuff repair by mediating tendon-derived stem cells. Article. *Regenerative Medicine*. 2021;16(4):359-372.
6. Gao R, Ye T, Zhu Z, et al. Small extracellular vesicles from iPSC-derived mesenchymal stem cells ameliorate tendinopathy pain by inhibiting mast cell activation. Article. *Nanomedicine*. 2022;17(8):513-529.
7. Gissi C, Radeghieri A, Lamorgese Passeri CA, et al. Extracellular vesicles from rat-bone-marrow mesenchymal stromal/stem cells improve tendon repair in rat Achilles tendon injury model in dose-dependent manner: A pilot study. Article. *PLoS ONE*. 2020;15(3)
8. Han Q, Wang S, Chen D, Gan D, Wang T. Exosomes derived from human umbilical cord mesenchymal stem cells reduce tendon injuries via the miR-27b-3p/ARHGAP5/RhoA signaling pathway. Article. *Acta biochimica et biophysica Sinica*. 2022;54(2):1-11.
9. Han L, Liu H, Fu H, Hu Y, Fang W, Liu J. Exosome-delivered BMP-2 and polyaspartic acid promotes tendon bone healing in rotator cuff tear via Smad/RUNX2 signaling pathway. Article. *Bioengineered*. 2022;13(1):1459-1475.
10. Hayashi Y, Yimiti D, Sanada Y, et al. The therapeutic capacity of bone marrow MSC-derived extracellular vesicles in Achilles tendon healing is passage-dependent and indicated by specific glycans. Article. *FEBS Letters*. 2022;596(8):1047-1058.
11. Huang Y, He B, Wang L, et al. Bone marrow mesenchymal stem cell-derived exosomes promote rotator cuff tendon-bone healing by promoting angiogenesis and regulating M1 macrophages in rats. Article. *Stem Cell Research and Therapy*. 2020;11(1)
12. Jenner F, Wagner A, Gerner I, et al. Evaluation of the Potential of Umbilical Cord Mesenchymal Stromal Cell-

Derived Small Extracellular Vesicles to Improve Rotator Cuff Healing: A Pilot Ovine Study. *The American journal of sports medicine*. 2023:3635465221145958-3635465221145958.
13. Li J, Yao Z, Xiong H, et al. Extracellular vesicles from hydroxycamptothecin primed umbilical cord stem cells enhance anti-adhesion potential for treatment of tendon injury. Article. *Stem Cell Research and Therapy*. 2020;11(1)
14. Li X, Su Z, Shen K, et al. Eugenol-Preconditioned Mesenchymal Stem Cell-Derived Extracellular Vesicles Promote Antioxidant Capacity of Tendon Stem Cells in Vitro and in Vivo. Article. *Oxidative Medicine and Cellular Longevity*. 2022;2022
15. Li Z, Li Q, Tong K, et al. BMSC-derived exosomes promote tendon-bone healing after anterior cruciate ligament reconstruction by regulating M1/M2 macrophage polarization in rats. Article. *Stem Cell Research and Therapy*. 2022;13(1)
16. Liu H, Zhang M, Shi M, et al. Adipose-derived mesenchymal stromal cell-derived exosomes promote tendon healing by activating both SMAD1/5/9 and SMAD2/3. Article. *Stem Cell Research and Therapy*. 2021;12(1)
17. Liu A, Wang Q, Zhao Z, et al. Nitric Oxide Nanomotor Driving Exosomes-Loaded Microneedles for Achilles Tendinopathy Healing. Article. *ACS nano*. 2021;15(8):13339-13350.
18. Ren Y, Zhang S, Wang Y, et al. Effects of purified exosome product on rotator cuff tendon-bone healing in vitro and in vivo. Article. *Biomaterials*. 2021;276
19. Shen H, Yoneda S, Abu-Amer Y, Guilak F, Gelberman R. Stem cell-derived extracellular vesicles attenuate the early inflammatory response after tendon injury and repair. *J orthop res*. 2019;
20. Shi Y, Kang X, Wang Y, et al. Exosomes derived from bone marrow stromal cells (BMSCs) enhance tendon-bone healing by regulating macrophage polarization. Article. *Medical Science Monitor*. 2020;26
21. Shi G, Wang Y, Wang Z, et al. A novel engineered purified exosome product patch for tendon healing: An explant in an ex vivo model. Article in Press. *Journal of orthopaedic research : official publication of the Orthopaedic Research Society*. 2020;
22. Song K, Jiang T, Pan P, Yao Y, Jiang Q. Exosomes from tendon derived stem cells promote tendon repair through miR-144-3p-regulated tenocyte proliferation and migration. Article. *Stem Cell Research and Therapy*. 2022;13(1)
23. Tao SC, Huang JY, Li ZX, Zhan S, Guo SC. Small extracellular vesicles with LncRNA H19 "overload": YAP Regulation as a Tendon Repair Therapeutic Tactic. Article. *iScience*. 2021;24(3)102200.
24. Wang C, Song W, Chen B, Liu X, He Y. Exosomes Isolated From Adipose-Derived Stem Cells: A New Cell-Free Approach to Prevent the Muscle Degeneration Associated With Torn Rotator Cuffs. Article. *The American journal of sports medicine*. 2019;47(13):3247-3255.
25. Wang Y, He G, Guo Y, et al. Exosomes from tendon stem cells promote injury tendon healing through balancing synthesis and degradation of the tendon extracellular matrix. Article. *Journal of Cellular and Molecular Medicine*. 2019;23(8):5475-5485.
26. Wang C, Tan J, Zhang Y, Chen D, He Y. In Situ-Forming Fibrin Gel Encapsulation of MSC-Exosomes for Partial-Thickness Rotator Cuff Tears in a Rabbit Model: Effectiveness Shown in Preventing Tear Progression and Promoting Healing. Article. *Journal of Bone and Joint Surgery*. 2022;104(16):1492-1502.
27. Wang C, Zhang Y, Zhang G, Yu W, He Y. Adipose Stem Cell-Derived Exosomes Ameliorate Chronic Rotator Cuff Tendinopathy by Regulating Macrophage Polarization: From a Mouse Model to a Study in Human Tissue. Article. *The American journal of sports medicine*. 2021;49(9):2321-2331.
28. Wang F, Qian J, Yang M-y, et al. MiR-6924-5p-rich exosomes derived from genetically modified Scleraxis-overexpressing PDGFR alpha(+) BMMSCs as novel nanotherapeutics for treating osteolysis during tendon-bone healing and improving healing strength. Article. *Biomaterials*. 2021;279121242.
29. Wang C, Hu Q, Song W, Yu W, He Y. Adipose Stem Cell-Derived Exosomes Decrease Fatty Infiltration and Enhance Rotator Cuff Healing in a Rabbit Model of Chronic Tears. Article. *The American journal of sports medicine*. 2020;48(6):1456-1464.
30. Wellings EP, Huang TCT, Li J, et al. Intrinsic Tendon Regeneration After Application of Purified Exosome Product: An In Vivo Study. Article. *Orthopaedic Journal of Sports Medicine*. 2021;9(12)
31. Wu XD, Kang L, Tian J, et al. Exosomes derived from magnetically actuated bone mesenchymal stem cells promote tendon-bone healing through the miR-21-5p/SMAD7 pathway. Article. *Materials Today Bio*. 2022;15100319.
32. Wu B, Chen H, Shi X, et al. Exosomes Derived from Bone Marrow Mesenchymal Stem Cell Preconditioned by Low-

Intensity Pulsed Ultrasound Stimulation Promote Bone-Tendon Interface Fibrocartilage Regeneration and Ameliorate Rotator Cuff Fatty Infiltration. *Medical Science Monitor*. 2021;

33. Xu T, Lin Y, Yu X, et al. Comparative Effects of Exosomes and Ectosomes Isolated From Adipose-Derived Mesenchymal Stem Cells on Achilles Tendinopathy in a Rat Model. Article. *The American journal of sports medicine*. 2022;50(10):2740-2752.
34. Xu H, Zhu Y, Hsiao AWT, et al. Bioactive glass-elicited stem cell-derived extracellular vesicles regulate M2 macrophage polarization and angiogenesis to improve tendon regeneration and functional recovery. Article. *Biomaterials*. 2023;294
35. Xu J, Ye Z, Han K, et al. Infrapatellar Fat Pad Mesenchymal Stromal Cell-Derived Exosomes Accelerate Tendon-Bone Healing and Intra-articular Graft Remodeling After Anterior Cruciate Ligament Reconstruction. Article. *The American journal of sports medicine*. 2022;50(3):662-673.
36. Yao Z, Li J, Wang X, et al. Microrna-21-3p engineered umbilical cord stem cell-derived exosomes inhibit tendon adhesion. Article. *Journal of Inflammation Research*. 2020;13:303-316.
37. Yao Z, Li J, Xiong H, et al. MicroRNA engineered umbilical cord stem cell-derived exosomes direct tendon regeneration by mTOR signaling. Article. *Journal of Nanobiotechnology*. 2021;19(1)
38. Ye T, Chen Z, Zhang J, et al. Large extracellular vesicles secreted by human iPSC-derived MSCs ameliorate tendinopathy via regulating macrophage heterogeneity. Article. *Bioactive Materials*. 2023;21:194-208.
39. Yu Y, Sun B, Wang Z, et al. Exosomes From M2 Macrophage Promote Peritendinous Fibrosis Posterior Tendon Injury via the MiR-15b-5p/FGF-1/7/9 Pathway by Delivery of circRNA-Ep400. Article. *Frontiers in Cell and Developmental Biology*. 2021;9
40. Yu H, Cheng J, Shi W, et al. Bone marrow mesenchymal stem cell-derived exosomes promote tendon regeneration by facilitating the proliferation and migration of endogenous tendon stem/progenitor cells. Article. *Acta Biomaterialia*. 2020;106:328-341.
41. Zhang X, Han Z, Han K, et al. Loading Mesenchymal Stem Cell-Derived Exosomes Into a Traditionally Designed Rotator Cuff Patch: A Potential Strategy to Enhance the Repair of Chronic Rotator Cuff Tear Associated With Degenerative Changes. Article. *The American journal of sports medicine*. 2022;50(8):2234-2246.
42. Zhang X, Li A, Han K, et al. Anti-inflammatory and Tendon-Protective Effects of Adipose Stem Cell-Derived Exosomes with Concomitant Use of Glucocorticoids. Article. *Stem Cells International*. 2022;2022
43. Zhang M, Liu H, Cui Q, et al. Tendon stem cell-derived exosomes regulate inflammation and promote the high-quality healing of injured tendon. Article. *Stem Cell Research and Therapy*. 2020;11(1)
44. Zhang T, Yan S, Song Y, et al. Exosomes secreted by hypoxia-stimulated bone-marrow mesenchymal stem cells promote grafted tendon-bone tunnel healing in rat anterior cruciate ligament reconstruction model. Article. *Journal of Orthopaedic Translation*. 2022;36:152-163.
45. Zhao H, Jiang H, Zhang H, et al. Adipose-Derived Mesenchymal Stem Cell-Derived Extracellular Vesicles Rescue Tendon Injury in Rat via the miR-19 a/IGFBP3 Axis. Article. *Stem Cells International*. 2022;2022
46. Zhu Z, Gao R, Ye T, et al. The Therapeutic Effect of iMSC-Derived Small Extracellular Vesicles on Tendinopathy Related Pain Through Alleviating Inflammation: An in vivo and in vitro Study. Article. *Journal of Inflammation Research*. 2022;15:1421-1436.
